# Prevalence, Risk Behaviors, and Virological Characteristics of Hepatitis B Virus Infection in a Group of Men Who Have Sex with Men in Brazil: Results from a Respondent-Driven Sampling Survey

**DOI:** 10.1371/journal.pone.0160916

**Published:** 2016-08-10

**Authors:** Marina P. Oliveira, Márcia A. D. Matos, Ágabo M. C. Silva, Carmen L. R. Lopes, Sheila A. Teles, Marcos A. Matos, Natália Spitz, Natalia M. Araujo, Rosa M. S. Mota, Ligia R. F. S. Kerr, Regina M. B. Martins

**Affiliations:** 1 Institute of Tropical Pathology and Public Health, Federal University of Goiás, Goiânia, Goiás, Brazil; 2 Faculty of Nursing, Federal University of Goiás, Goiânia, Goiás, Brazil; 3 Oswaldo Cruz Institute, Oswaldo Cruz Foundation, Rio de Janeiro, Rio de Janeiro, Brazil; 4 Department of Community Health, Federal University of Ceará, Fortaleza, Ceará, Brazil; Fudan University, CHINA

## Abstract

**Background:**

Men who have sex with men (MSM) are at increased risk of exposure to hepatitis B virus (HBV) compared with the general population. This study aims to assess the epidemiological and virological characteristics of HBV infection in a sample of MSM in Brazil, where data are scarce.

**Methods:**

A cross-sectional study was conducted among MSM in the City of Goiânia, Central Brazil, from March to November 2014, using Respondent-Driven Sampling (RDS). After signing the consent form, participants were interviewed and a blood sample collected. All samples were tested for HBV serological markers and HBV DNA. HBV nucleotide sequence analysis was also performed.

**Results:**

A total of 522 MSM were recruited in the study. The prevalence of HBV infection (current or past [presence of anti-HBc marker]) was 15.4% (95% CI: 8.7–25.8) and the rate of HBsAg carriers was 0.6% (95% CI: 0.2–1.6). About 40% (95% CI: 32.3–48.8) of the participants had serological evidence of previous HBV vaccination (reactive for isolated anti-HBs). In addition, 44.3% (95% CI: 36.1–52.9) were seronegative for all HBV markers. Age over 25 years old, receptive anal intercourse, previous sex with women, and history of sexually transmitted infections (STIs) were factors associated with HBV infection. HBV DNA was detected only in HBsAg-positive individuals. HBV isolates were classified into genotype A (subgenotypes A1 and A2), and some mutations were identified throughout the genome. Therefore, occult HBV infection was not observed in the study population.

**Conclusions:**

Public health strategies should be improved for the MSM population in order to prevent HBV and other STIs, as well as to provide appropriate management of patients with active infections.

## Introduction

Hepatitis B virus (HBV) infection has a wide global distribution. The World Health Organization (WHO) estimates that there are over 240 million chronically infected people worldwide. The chronic form of this infection is associated with a variety of clinical manifestations, ranging from an asymptomatic carrier state to severe liver disease, including cirrhosis and hepatocellular carcinoma (HCC) [1].

The traditional serological marker of hepatitis B is the HBV surface antigen (HBsAg), which is detectable in serum during acute and chronic infection. However, with the development of highly sensitive molecular biology techniques, HBV DNA has been detected in serum and/or liver without detectable HBsAg. This profile is called occult HBV infection (OBI) and has been associated with hepatitis B reactivation, increased severity of liver disease, and HCC development. In addition, an increased risk of HBV transmission through blood transfusion, organ transplantation, and hemodialysis due to OBI has been reported [2,3].

HBV isolates are classified into ten genotypes, designated A-J, based on sequence divergence greater than 7.5% in the complete genome. Most genotypes further segregate into subgenotypes that differ from each other by 4–7.5%. HBV genotypes/subgenotypes have different geographical distributions, and influence clinical outcomes and response to treatment [4,5]. In Brazil, genotype A is the most prevalent, followed by genotypes D and F, with a higher proportion of subgenotype A1 [6,7].

The genetic variability of HBV can lead to the occurrence of different diagnosis and clinical profiles. Mutations in the Pre-S/S gene region have been linked to low HBsAg levels and HBV vaccine/immunoglobulin escape, whereas those found in the Pre-C/C and BCP (basal core promoter) gene regions can lead to decreased hepatitis B e antigen (HBeAg) expression and to progression to severe liver disease [8,9].

HBV infection occurs in a considerable number of men who have sex with men (MSM). According to the US CDC [10], about 20% of new cases of HBV infection in adults in the United States of America occur among gay and bisexual men. In addition, a high rate of chronic hepatitis B was reported in MSM [11]. Some studies have shown that MSM are at high risk of sexually transmitted infections (STIs), including hepatitis B [12–18]. This greater vulnerability involves the context of violence, conditions of sexual practices such as unprotected anal intercourse and multiple sexual partners in addition to low access to health services and social integration, which can lead to unsafe sexual practices [13,17].

Worldwide, HBV infection prevalence in MSM varies according to geographical region as well as with the characteristics of the selected subgroup studied. Thus, the prevalence of this infection in MSM has ranged from 0.9% in Lebanon [19] to 44% in the Netherlands [11]. In addition, higher HBV rates have been described in specific subgroups of MSM, such as gays and bisexuals homeless young adults in USA (52.4%) [20], HIV-positive MSM in Taiwan (52.9%) [16] and male transvestite commercial sex workers in Uruguay (50.5%) [21].

In Brazil, previous [22,23] and current [15] studies concerning HBV prevalence among MSM are still rare, and no data regarding the molecular epidemiology of HBV in MSM has been published thus far. Therefore, this study was conducted in order to investigate the HBV prevalence, associated factors, occult infection rate and molecular characterization of viral isolates in MSM in Central Brazil.

## Material and Methods

### Study population and sampling method

A cross-sectional study among MSM was conducted in Goiânia (1,256,514 inhabitants), the capital of the State of Goiás in Central Brazil. From March to November 2014, a total of 522 MSM were recruited using Respondent-Driven Sampling (RDS), a chain-referral sampling method in which initial participants, called seeds, recruit a limited number of additional participants. These participants recruit more participants and this process goes on until the desired sample size is reached. This recruitment strategy is an efficient data collection method and it is useful to study hidden populations such as MSM [24,25]. Initially, formative research was conducted to determine study logistics and to select the seeds. Based on their extensive social network, three seeds were first selected by collaborating with governmental and non-governmental organizations supporting LGBT (Lesbian, Gay, Bisexual and Transgender) individuals and during the gay pride parade. Additionally, two more seeds were included during the study to enroll additional participants. Each seed received three numbered referral coupons to invite other eligible MSM to the study (first wave). Eligibility criteria for participation were being born male sex, aged 18 years or older, leaving in the Goiânia metropolitan area, reporting have had sex with another man in the 12 months preceding the study, do not identify as transsexual, and presenting a valid recruitment coupon.

Considering that MSM is a hard-to-reach population, the sample size calculation took into consideration the expected design effect of 2.0 [26]. The minimum sample size of 418 participants was calculated using an estimated prevalence for exposure to HBV of 11.4% [15], with confidence interval of 95% and a precision of 4.4%. Due to the return of some coupons previously issued, the sample size reached 522.

After being informed of the objectives and methodology of the study, individuals signed the consent form and answered an interviewer-administered structured questionnaire to collect information about their personal network size, relationship with the recruiter, sociodemographic characteristics and possible factors associated with HBV infection. Then, a blood sample was collected from each participant for laboratory testing. All participants received three referral coupons to recruit new MSM of their social relationships (friends and/or sexual partners), in addition to educational materials and condoms. As an incentive, each participant received four public transportation tickets and two additional transportation tickets for each participant they recruited into the study.

The procedures of this study were approved by the Ethics Committee of the Federal University of Goiás (UFG), Goiânia, Goiás, Brazil. All participants signed informed written consent. This consent form was approved by the ethics committee.

### Serological tests

Serum samples were tested by enzyme-linked immunosorbent assay (ELISA) for detection of HBsAg (Hepanostika HBsAg Ultra, Biomérieux, Boxtel, The Netherlands), antibodies against hepatitis B core antigen (anti-HBc) (Hepanostika anti-HBc Uni-form, Biomérieux), and antibodies against HBsAg (anti-HBs) (Bioelisa anti-HBs, Biokit, Barcelona, Spain). HBsAg reactive samples were subsequently tested for the presence of anti-HBc IgM (Bioelisa anti-HBc IgM, Biokit), HBeAg and antibodies against HBeAg (anti-HBe) (Eti-Ab/Ebk Plus, Diasorin, Italy).

### Molecular tests

DNA was extracted from all samples using phenol-chloroform method [27]. The Pre-S/S region was amplified by semi-nested polymerase chain reaction (PCR) [28]. HBV DNA-positive samples were submitted to amplification of the complete genome [29]. When the full-length genome amplification was not successfully achieved, the S, BCP, and Pre-C/C gene regions were further amplified, as previously described [28]. Nucleotide sequences of the amplified regions were determined by direct sequencing using a BigDye Terminator 3.1 cycle sequencing kit (Applied Biosystems, Foster City, CA), on an ABI 3130 automated sequencer (Applied Biosystems). Sequences were aligned and edited using SeqMan II v.5.01 (DNASTAR), Clustal W and BioEdit. HBV genotypes and subgenotypes were determined by phylogenetic analysis using MEGA program v.6.0 (Molecular Evolutionary Genetics Analysis) and published reference sequences available in GenBank database (http://www.ncbi.nlm.nih.gov/). To identify mutations in the HBV genome, the deduction of amino acids (aa) was performed from nucleotide sequences by using MEGA program v.6.0. The nucleotide sequences obtained in this study were deposited in GenBank under the accession numbers KU900750 to KU900754.

### Data analysis

Prevalence and 95% confidence intervals (CI) were estimated using RDS Analysis Tool (RDSAT) v.5.6 (http://rds-analysis-tool.software.informer.com/versions/). To reduce possible biases associated with chain referral sampling, RDSAT provides weights for each participant based on his social network size and recruitment patterns [24]. Data and weights generated through RDSAT were exported to SPSS v.20 (SPSS Inc., IBM, Chicago, US) for weighted analysis of variables associated with HBV infection [current or past (defined by the presence of anti-HBc serological marker)] using Pearson’s chi-square test and Fisher’s exact test. Variables associated with this infection at p≤0.10 in the univariate analysis were included in the multivariable analysis using a logistic regression model. Finally, a p-value less than 0.05 was considered statistically significant. The design of social networks to visualize the distribution of the HBV exposure in the recruitment network was performed using NetDraw software (http://www.analytictech.com/downloadnd.htm).

## Results

### Sampling

Of a total of 1,227 coupons issued, 530 (43.2%) were redeemed. Among the 530 MSM who presented a valid recruitment coupon, eight were not selected (two refused to give a blood sample and six were under 18 years old). Therefore, 522 MSM were included in the analysis (5 seeds and 517 recruits). The median number of waves by seed was 9 (range 3–15), and the median number of recruits by seed was 103 (range 15–169).

### Characteristics of participants

The sociodemographic characteristics of the study population are shown in Table 1. Participants were mostly young (≤25 years old, 60%). The majority self-identified as gay (74.9%), followed by bisexual (19.4%) and transvestite (5.7%). Skin color was assessed by self-report (59% claimed brown or mixed *(pardo)*, 18.9% white, 16.6% black and 5.6% others). Most of MSM were single (76.9%), previously attended high school (10–12 years of study, 63.9%) and were in the lowest tier of Brazilian social class (61.3%).

**Table 1:** Sociodemographic characteristics of 522 MSM in Goiânia, Central Brazil.

Characteristics	N	Unweighted %	Weighted %	95% CI
Age (median: 23.6; range: 18–62)				
≤ 25 years	338	64.75	60.0	50.8–68.5
> 25 years	184	35.25	40.0	31.5–49.2
Self-identification				
Gay	355	68.0	74.9	67.2–81.4
Bisexual	76	14.6	19.4	13.4–27.2
Transvestite	91	17.4	5.7	3.8–8.3
Skin color/race				
Brown (*pardo*)	296	56.7	59.0	50.0–67.4
White	103	19.7	18.9	13.5–25.7
Black	91	17.4	16.6	9.9–26.4
Yellow (Asian)	21	4.0	4.1	1.7–9.2
Indigenous	11	2.1	1.5	0.7–3.4
Marital status				
Single	420	80.5	76.9	66.9–84.5
Married/in stable relationship	94	18.0	20.1	12.9–9.9
Separated/divorced	8	1.5	3.1	0.9–9.4
Education (years) (n = 520)^a^				
≤ 9	77	14.8	13.7	9.0–20.3
10–12	307	59.0	63.9	55.9–71.3
≥ 13	136	26.2	22.4	17.0–28.9
Social class (Brazilian criteria)^b^				
A/B (≥ R$ 8,641)	7	1.4	0.4	0.1–1.7
C (R$ 2,005–8,640)	81	15.5	9.9	6.6–14.5
D (R$ 1,255–2,004)	140	26.8	28.4	20.3–38.3
E (≤ R$ 1,254)	294	56.3	61.3	52.1–69.7

MSM, men who have sex with men; CI, confidence interval; R$, Brazilian real.

^a^Missing data are not shown.

^b^Brazilian criteria classifies individuals into five economic classes [30]: “A” constitutes the highest tier of social class, while “E” the lowest one (US$ 1.00 is approximately equal to R$ 2.32).

### Prevalence of HBV serological markers

**Fig 1:**
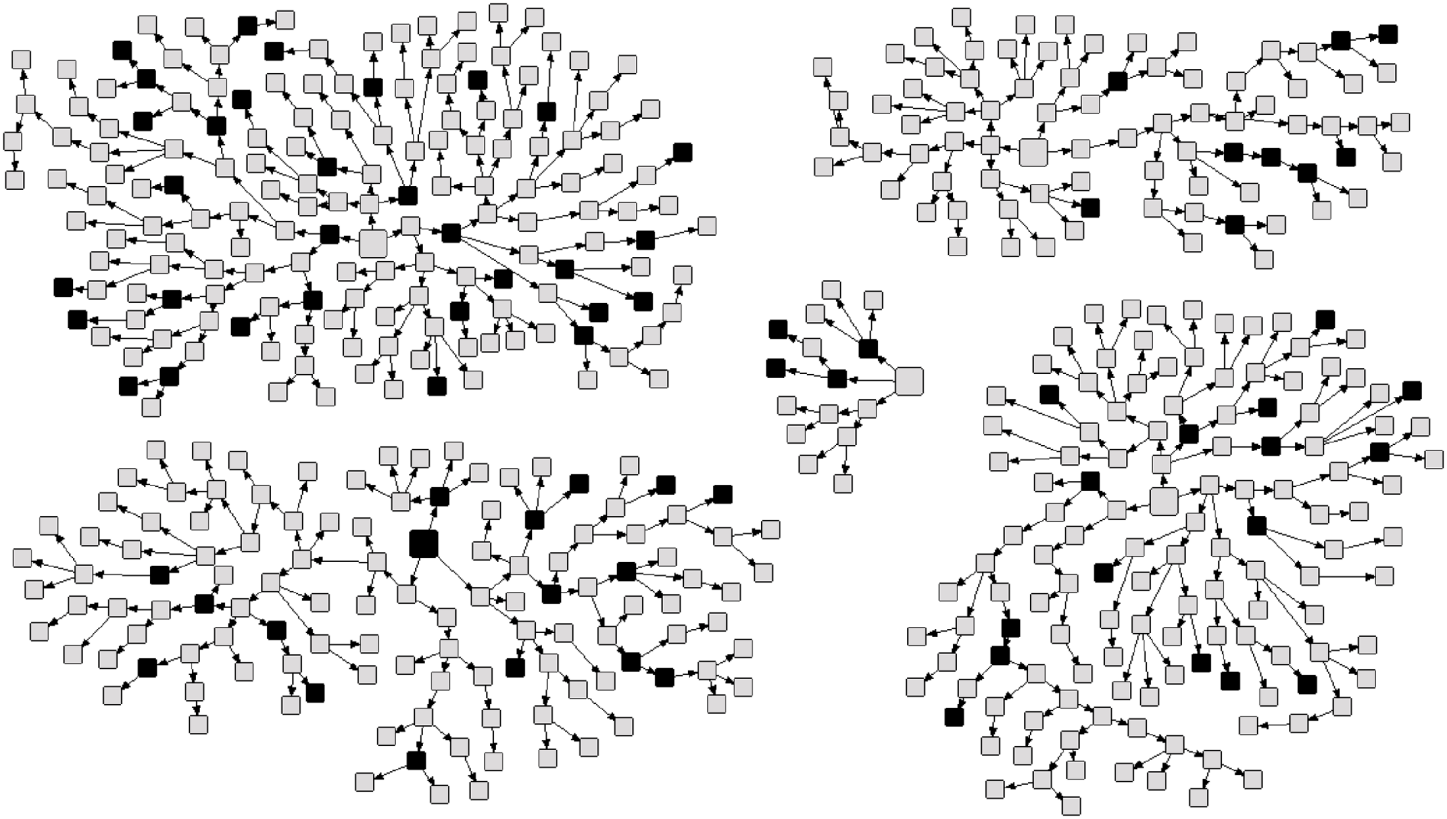
Recruitment networks of 522 men who have sex with men in Goiânia, Central Brazil. The seeds are indicated by large squares and recruits by small squares. Individuals who had been exposed to HBV are in black and others in gray.

Table 2 shows the prevalence of HBV serological markers. Among the 522 MSM studied, 77 (15.4%; 95% CI: 8.7 to 25.8) had been exposed to HBV. Of these, 5 (0.6%) were anti-HBc/HBsAg carriers, 60 (9.3%) were reactive for anti-HBc/anti-HBs and 12 (5.5%) were positive for anti-HBc only. Moreover, 206 (40.3%) were reactive for isolated anti-HBs. In addition, 239 (44.3%) were seronegative for all hepatitis B serological markers. The representation of the recruitment network indicating cases of exposure to HBV is presented in Fig 1.

**Table 2:** Prevalence of HBV serological markers among 522 MSM in Goiânia, Central Brazil.

HBV markers	Total	Unweighted %	Weighted % (95% CI)
Anti-HBc/ HBsAg	5	1.0	0.6 (0.2–1.6)
Anti-HBc/ anti-HBs	60	11.5	9.3 (5.9–14.5)
Anti-HBc only	12	2.3	5.5 (1.2–21.8)
Any exposure marker	77	14.8	15.4 (8.7–25.8)
Anti-HBs (immunized)	206	39.5	40.3 (32.3–48.8)
Absence of any marker (susceptible)	239	45.8	44.3 (36.1–52.9)

HBV, hepatitis B virus; MSM, men who have sex with men; CI, confidence interval; HBsAg, hepatitis B surface antigen; anti-HBc, antibodies against hepatitis B core antigen; anti-HBs, antibodies against HBsAg.

### Factors associated with HBV infection

Univariate analysis of demographic, behavioral characteristics, and HIV serological status among unvaccinated MSM (Table 3) revealed that age over 25 years, more than 10 sex partners in lifetime, history of drugs or alcohol use during sex, receptive anal intercourse, previous sex with women and history of STIs were statistically associated with HBV infection (p<0.05). These variables in addition to ever having received payment for sex (p = 0.055) and HIV seropositivity (p = 0.079) were included in a multivariate analysis using a logistic regression model. Age over 25 years old (p<0.001), receptive anal intercourse (p = 0.001), previous sex with women (p = 0.038), and history of STIs (p = 0.001) were independent factors associated with HBV infection in the study population (Table 4).

**Table 3:** Demographic, behavioral characteristics and HIV serological status associated with HBV infection among unvaccinated MSM in Goiânia, Central Brazil.

Variable	HBV Pos./total	Unweighted %	Weighted % (95% CI)	OR (95% CI)	P value
Age (years)					
≤ 25	22/183	12.0	5.2 (2.4–10.8)	1	
> 25	55/133	41.3	46.3 (28.1–65.5)	15.73 (5.10–48.39)	0.000
Self-identification					
Gay	49/207	23.7	28.0 (14.8–46.6)	1.96 (0.48–8.02)	
Bisexual	17/61	27.9	27.7 (12.7–50.4)	1.94 (0.43–8.78)	0.493
Transvestite	11/48	22.9	16.5 (5.9–38.4)	1	
Number of sexual partners in lifetime					
≤ 10	16/93	17.2	11.9 (5.9–22.6)	1	
> 10	61/223	27.3	35.1 (19.3–55.0)	3.99 (1.30–12.23)	0.012
Ever used drugs or alcohol during sex					
No	20/102	19.6	13.6 (7.0–24.8)	1	
Yes	57/214	26.6	33.4 (18.0–53.4)	3.19 (1.10–9.65)	0.034
Ever received payment for sex					
No	45/173	26.0	17.6 (11.5–26.1)	1	
Yes	32/143	22.4	41.5 (18.3–69.3)	3.32 (0.94–11.69)	0.055
Receptive anal intercourse					
No	11/57	19.3	7.0 (2.6–17.5)	1	
Yes	66/259	25.5	32.3 (18.9–49.5)	6.35 (1.80–22.53)	0.002
Condom use at anal intercourse					
Always	30/157	19.1	22.5 (9.0–46.1)	1	
Not Always	47/159	29.5	31.3 (18.9–47.2)	1.57 (0.44–5.62)	0.484
Sex with women					
No	32/169	18.9	15.2 (9.4–23.7)	1	
Yes	45/147	30.6	34.3 (17.3–56.6)	2.91 (1.00–8.48)	0.045
Group sex					
No	28/142	19.7	26.3 (11.2–50.1)	1	
Yes	49/172	28.5	26.5 (15.4–41.5)	1.01 (0.29–3.49)	0.987
Sex against will					
No	57/216	26.4	24.2 (12.2–42.4)	1	
Yes	19/98	19.4	32.1 (15.4–55.0)	1.48 (0.42–5.24)	0.543
STIs					
No	44/232	18.9	12.0 (7.4–18.8)	1	
Yes	33/82	40.2	57.7 (31.9–79.9)	10.02 (3.04–33.08)	<0,001
HIV status					
Negative	54/246	21.9	20.6 (9.2–39.9)	1	
Positive	23/70	32.8	45.2 (24.4–67.8)	3.17 (0.84–11.93)	0.079
Ever used any illicit drug					
No	35/155	22.6	23,6 (14,2–36,6)	1	
Yes	42/160	26.2	26.5 (13.4–45.5)	1.17 (0.41–3.32)	0.772
Blood transfusion lifetime					
No	70/294	23.8	25.5 (14.4–41.0)	1	
Yes	6/17	35.3	31.3 (9.4–66.6)	1.33 (0.26–6.87)	0.117

HBV, hepatitis B virus; MSM, men who have sex with men; CI, confidence interval; OR, odds ratio; STIs, sexually transmitted infections; HIV, human immunodeficiency virus.

**Table 4:** Multivariate analysis of factors associated with HBV infection among MSM in Goiânia, Central Brazil.

Variable	Adjusted OR (95% CI)^a^	P value
Age (years)		
≤ 25	1	
> 25	11.32 (4.08–31.42)	<0.001
Number of sexual partners in lifetime		
≤ 10	1	
> 10	1.68 (0.52–5.45)	0.386
Ever used drugs or alcohol during sex		
No	1	
Yes	1.65 (0.61–4.47)	0.321
Ever received payment for sex		
No	1	
Yes	0.81 (0.27–2.44)	0.702
Receptive anal intercourse		
No	1	
Yes	11.60 (2.60–51.72)	0.001
Sex with women		
No	1	
Yes	2.83 (1.06–7.56)	0.038
STIs		
No	1	
Yes	5.79 (2.16–15.56)	0.001
HIV status		
Negative	1	
Positive	2.01 (0.75–5.42)	0.164

HBV, hepatitis B virus; MSM, men who have sex with men; CI, confidence interval; STIs, sexually transmitted infections; HIV, human immunodeficiency virus; ^a^Adjusted odds ratio for the following variables: age, number of sexual partners in lifetime, use of drugs or alcohol during sex, received payment for sex, receptive anal intercourse, sex with women, STIs and HIV.

### Characteristics of HBV DNA positive samples

Of the 522 samples, HBV DNA was detected only in the five anti-HBc/HBsAg positive ones. Thus, occult HBV infection was not observed in the study population. Relative to HBV DNA positive samples (Table 5), two and three were HBeAg and anti-HBe reactive, respectively. Anti-HBc IgM marker was not detected in any of these samples. Two samples (Y431 and Y494) were successfully amplified for full-length HBV genome, one (Y513) for Pre-S/S, BCP and Pre-C/C, one (Y02) for Pre-S/S, and one (Y413) for S gene region. Sequence analysis of these samples revealed that all HBV isolates had the T131N amino acid substitution in the S gene. In addition, analysis of BCP and Pre-C/C gene regions showed the double mutation A1762T/G1764A in sample Y431, G1862T/G1888A in Y494, and G1862T in Y513. No mutations were detected in X and overlapping HBV polymerase gene regions.

**Table 5:** Hepatitis B virus biomarkers in MSM with HBV DNA positive in Goiânia, Central Brazil.

HSH	Age (years)	Serological markers		Genome/ regions amplified	Mutations		Subgenotype
		HBeAg	Anti-HBe		S region	BCP and Pre-C/C region	
Y02	37	+	-	Pre-S/S	T131N	-	A2
Y413	39	-	+	S	T131N	-	A1
Y431	52	-	+	Full genome	T131N	A1762T, G1764A	A2
Y494	37	+	-	Full genome	T131N	G1862T, G1888A	A1
Y513	26	-	+	Pre-S/S, BCP and Pre-C/C	T131N	G1862T	A1

HBV, hepatitis B virus; MSM, men who have sex with men; HBeAg, hepatitis B e antigen; anti-HBe, antibodies against HBeAg; BPC, basal core promoter.

**Fig 2:**
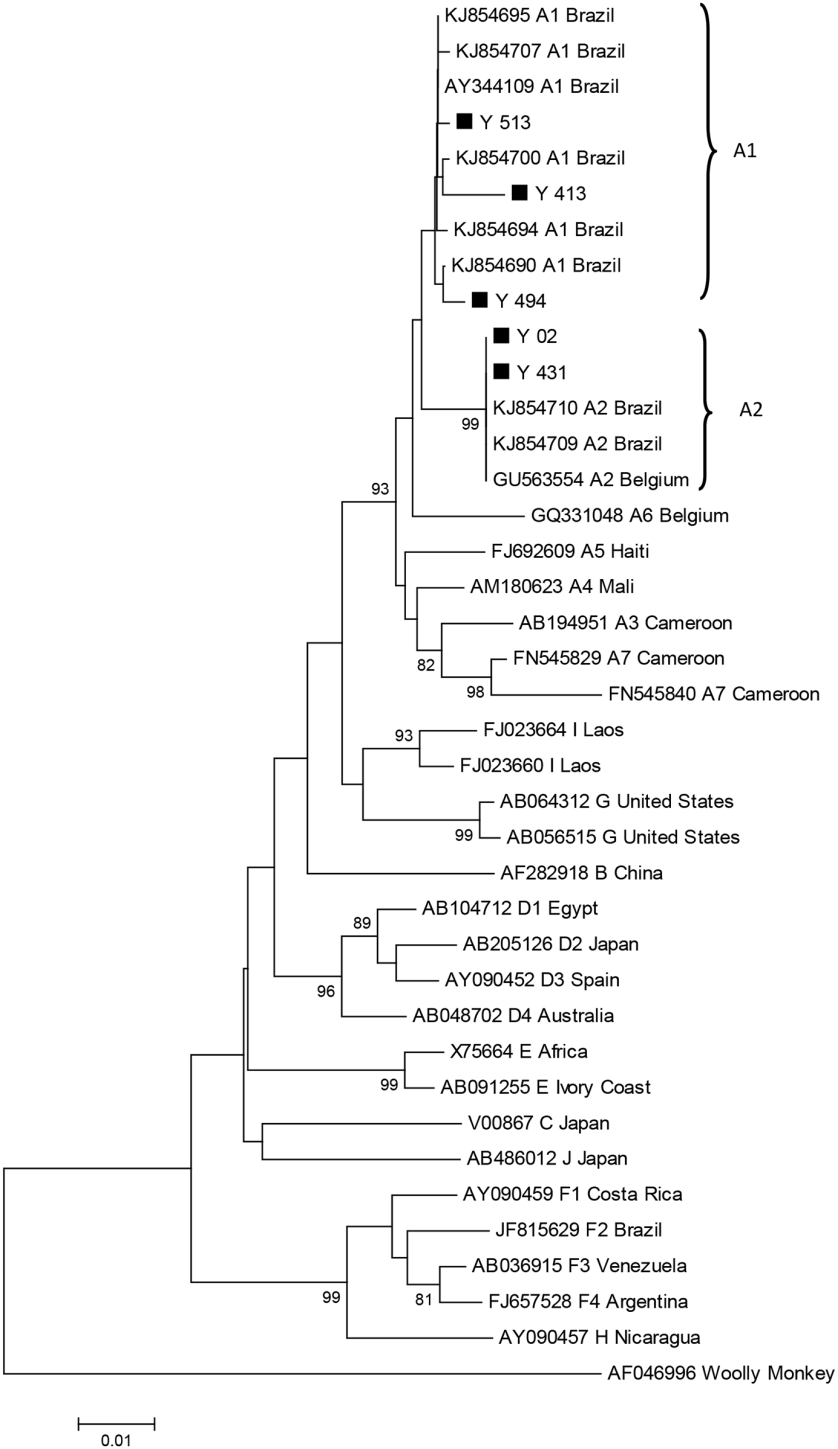
Phylogenetic tree analysis of the S region of hepatitis B virus (HBV). The phylogenetic tree performed by using the CLUSTAL W program and analyzed by Kimura two-parameter methods. Genetic distances were calculated by the maximum composite likelihood. Phylogenetic tree was constructed by the neighbor-joining method using MEGA v.6.0 software (bootstrap resampling test with 1,000 replicates), including 5 isolates from Brazilian MSM (black square), 33 GenBank sequences of genotypes A–J (GenBank accession number, HBV genotype, and country of origin are indicated) and Wooly Monkey HBV (AF046996) was used as the out group.

Phylogenetic analysis of the S gene showed that the five isolates belonged to HBV genotype A, subgenotypes A1 (n = 3) and A2 (n = 2) (Fig 2). These results were further confirmed by analysis of other amplified genomic regions.

## Discussion

To our knowledge, this is the first study to investigate the epidemiological and virological aspects of HBV infection in MSM in Brazil using RDS as a recruitment and data analysis method. The sociodemographic characteristics of the studied population were similar to previous reports for Brazilian MSM, such as age under 25 years old [15], self-identification as brown [31], single status [31], high school attended, and lowest tier of Brazilian social class [15,32]. Although these characteristics were similar to those reported previously, they can also be influenced by RDS. This recruitment method of participants depends on a connected social network, and like other sampling methods for hidden populations, there are tradeoffs [25,33].

The prevalence of HBV infection (15.4%; 95% CI: 8.7–25.8) estimated in this study was similar to those found in MSM in Argentina (22.9%; 95% CI: 19.9–26.9) [34], Australia (10.6%; 95% CI: 10.0–11.4) [35], and the USA (19.4%; 95% CI: 15.0–24.0) [36]. These are countries with a low endemicity for this infection, similar to that reported in Brazil [37]. This prevalence was also similar to that obtained in another recent study among MSM in Brazil (Campinas City, São Paulo State: 11.4%; 95% CI: 7.8–14.9) [15]. Nevertheless, the prevalence determined in this study was higher than that observed in the general population in Central Brazil (5.3%; 95% CI: 4.6–6.1) [38], suggesting that MSM still have a greater vulnerability to HBV acquisition.

Moreover, the prevalence of anti-HBs alone showed that about 40% of the population had serological evidence of immunization against HBV. In addition, a high percentage (44.3%) of MSM was seronegative for all hepatitis B serological markers, indicating susceptibility to HBV infection. These data suggest lower vaccination coverage than desired, since the HBV vaccine is recommended and available free to all MSM in Brazil. Therefore, it is important to emphasize the need to reassess and improve strategies for prevention and control of hepatitis B in this group, mainly to increase the number of MSM vaccinated against HBV.

As described by other authors [14,36,39], multivariate analysis shows that age over 25 years old was independently associated with HBV infection in this study. The association between HBV infection and older age has been widely reported and it is probably due to the increased risk of exposure over time and also to the greater vaccination coverage especially among children and young people in the last decades [36,40].

Receptive anal intercourse and history of STIs were factors associated with HBV infection in this population, as well as in other MSM [14,39]. Indeed, mucosal lesions caused by anal sex have been associated with transmission of several STIs [41]. Additionally, sex with women was associated with HBV infection in this study. Although most participants have self-identified as gay, almost half (47%) reported previous sex with women. Studies indicate that men who have sex with both genders are a potential bridge for STIs between MSM and women [34]. A previous study conducted among Brazilian MSM has suggested that bisexual men who have practiced unprotected vaginal and anal sex with their female partners as well as unprotected anal intercourse with their male partners probably have an increased risk for HIV exposure and, consequently, for other STIs [23].

HBV DNA was found in all HBsAg-positive MSM, showing that they have active hepatitis B and a higher potential for HBV transmission. Additionally, this risk is directly related to the level of HBV DNA in serum, and it is usually higher in those HBeAg-positive patients [41,42].

Despite the importance of researching occult HBV infection due to the risk of progression to serious liver disease and potential transmission of HBV [2], there is only one study concerning to OBI in MSM in the world, showing an OBI prevalence of 0.2% in HIV-positive individuals in Germany [41]. Reflecting this low prevalence, OBI was not observed in MSM analyzed in this study.

Phylogenetic analysis showed that HBV genotype A (A1 and A2) was identified in the study population, corroborating previous studies which have indicated that this genotype is the most prevalent in Brazil [7,38]. In addition, all HBV isolates analyzed here presented the T131N substitution in the S gene. It has been suggested that this mutation is a natural polymorphism in HBV genotypes A and G, being associated with persistence of the HBV even after loss of HBsAg and anti-HBs seroconversion as well as with vaccine escape [43].

The typical double mutation in BCP (A1762T and G1764A) found in one HBeAg-negative individual (Y431) is responsible for decreased HBeAg expression and has been linked to HBV oncogenesis [9]. In this study, the Pre-C G1862T mutation was found in one HBeAg-negative (Y513) and in one HBeAg-positive (Y494) participant (along with G1888A), both belonging to subgenotype A1. As reported elsewhere [9,44], the presence of G1862T in some HBeAg-positive patients suggests that this mutation can be genotype specific, since it was predominantly found in HBV/A1 isolates. G1888A is also characteristic of subgenotype A1 [45]. These findings indicate that HBV genotypes/subgenotypes may display different clinical implications on the variability of BCP, Pre-C/C and S gene regions and may impact hepatitis B prognosis.

These results must be considered in the context of the study's limitations. First, as this was a cross-sectional study, it could not establish the causality between associated factors and HBV infection; therefore a longitudinal study is needed to explore these factors among Brazilian MSM. Second, as interviews were conducted face-to-face, it is possible that behavioral responses may be sensitive for some participants resulting in information biases, although interviewers were trained to minimize these biases. Third, RDS is a relatively recent method and has its own limitations regarding calculation of the sample size and appropriate tools for data analysis, as well as definition of estimators [46]. In this study, although we have considered a design effect of 2 to the sample size calculation, as often recommended in the literature [26,33], and some findings on multivariate associations were significant, CIs were wide due to the modest sample size. Lastly, the incentive reward offered by the RDS may attract more low-income MSM to participate in the study. In addition, this was also limited to MSM aged 18 years or older, potentially limiting the representativeness of the studied sample. Despite these limitations, RDS is an effective approach to access hidden populations such as MSM. Additionally, this study provided a comprehensive investigation of the epidemiological and virological characteristics of HBV in a group of MSM in Brazil. Therefore, further national study is necessary to confirm our findings.

In conclusion, this study shows that the HBV prevalence among MSM was higher than that previously reported in the general population in Central Brazil, and was associated with sexual risk behaviors. The large proportion of the study population showed susceptibility to HBV infection, highlighting the need to increase HBV immunization coverage in MSM, as well as the sexual health education programs in Brazil. It is also worth mentioning the need for expert assistance and monitoring of HBV DNA-positive individuals to prevent progression to more severe diseases.
